# What can rare variant genetics tell us about cognition and intellectual difficulties?

**DOI:** 10.1007/s00415-016-8326-6

**Published:** 2016-11-07

**Authors:** Rhys H. Thomas, Neil P. Robertson

**Affiliations:** Institute of Psychological Medicine and Clinical, Neurosciences Cardiff University, Cardiff, CF14 4XW UK

This month we discuss three types of rare genomic variation and specifically how rare variants impact on cognition and behaviour. Each of the highlighted studies employs a different outcome measure: diagnosis of autism spectrum disorder or intellectual difficulties; performance on cognitive testing; and the number of years in education. All harness the extreme power of massively parallel sequencing or genomic arrays together with innovative bioinformatic analyses. The first study identifies ‘ultra-rare variation’, these are variants that are never, or almost never, seen in the unaffected population. The second identifies larger areas of rearranged genomic DNA which change the gene dosage for one or many genes, termed copy number variants. These variants may cluster in previously recognised sites or they may exist idiosyncratically. The third study uses a strategy to focus on variation at the loci that vary most between us and other species and are, therefore, thought to be of special significance for identifying what defines the human species; communication and civilised behaviour. Genomic areas that are highly divergent are known as human-accelerated regions (HARs) and damaging mutations in these areas may be more likely to cause a cognitive or behavioural phenotype.

## Ultra-rare disruptive and damaging mutations influence educational attainment in the general population

Ganna and colleagues set out to identify ultra-rare genetic variants and explore whether the presence of these variants in highly constrained genes was associated with poorer educational performance. They had access to 14,133 individuals from Sweden, Estonia, and Finland; 5047 of whom had schizophrenia. They initially identified demographic associations with the number of years of education; lower in Finnish populations, lower in men, and lower in older people. They identified the ultra-rare variants from either exome or the genic component of genome sequencing and focussed only on highly conserved genes identifying disrupting mutations in 25%; damaging variants in 24%; and synonymous variants not predicted to change the protein in 78%.

Each disruptive or damaging mutation reduced the mean number of years of education by 3 months. Each subsequent disruptive or damaging mutation reduced the chances of attending college by 14%. These associations remained when 34% of the cohort with schizophrenia were analysed separately. The impact on years of education was more than doubled when highly conserved genes enriched for brain expression were the sole focus. They then set about confirming prior genomic associations with educational attainment. The presence of a pathogenic copy number variant reduced the time in education by 7.6 months. This was a large effect but not seen frequently (1.34% of the sample). In contrast, modelling the polygenic risk score could explain 2.9% of the variability in educational attainment, compared to 0.4% for the ultra-rare variants. This study joins a nascent body of evidence highlighting the importance of ultra-rare variation in the genetic architecture of common diseases alongside common variants and pathogenic copy number variants.

*Ganna A. et al. (2016) Nat Neurosci. (Epub ahead of print) *doi:10.1038/nn.4404.

## Cognitive performance among carriers of pathogenic copy number variants: analysis of 152,000 UK Biobank subjects

The UK Biobank is a cohort collection of half a million older adult volunteers, both with and without disease. In addition to donating DNA for analysis, they undergo a number of physical and cognitive tests. Analysis of the first available data set of 152,728 adults enabled Kendall and colleagues to produce a map of copy number variants. Limiting themselves to a set of 93 copy number variants that are strongly associated with disease, they confirmed that population frequencies were similar to that seen in smaller control samples elsewhere. The most common copy number variant, 15q11.2, was found in 0.5% of the cohort.

They then examined the results of cognitive function testing of participants, comparing the unaffected majority with 1087 individuals who had one of the 12 copy number variants associated with schizophrenia, and 484 who had one of the 41 associated with other neurodevelopmental disorders. Copy number variant carriers performed less well on all 14 tests, with 9 of these surviving statistical corrections for multiple testing. Both groups with copy number variants were less likely to go to college or University or achieve examinations aged 18 (A levels). Those with copy number variants were also more likely to have jobs that require less training or fewer academic qualifications. This study confirms prior associations that even in those ‘healthy’ individuals with a pathogenic copy number variant, cognitive performance is impaired. These data are of importance for genetic counsellors who have the unenviable task of advising families on these rare genomic mutations with pleotropic effects.

*Kendall K. et al. (2016) Biol Psychiatry (Epub ahead of print)* doi:10.1016/j.biopsych.2016.08.014

## Mutations in human-accelerated regions disrupt cognition and social behaviour

Most HARs are within close proximity of a gene (within 1 Mb) and it is proposed that they act as transcriptional enhancers. Until now, only circumstantial evidence linked mutations in HARs to neurological disorders. In this study, investigators first identified 2737 HARs in six published papers. The HARs were twice as likely to not carry rare variants, suggesting that they are both critical and conserved. Prediction tools were then used to look at gene expression within HAR regions. HARs show significant enrichment for foetal and adult brain profiles (*p* < 0.0001), suggesting a preference for brain activity. Over 80% of HARs overlapped areas of active transcription, promoters, or enhancers. This included 45% in neural samples of which many were tissue specific, including 168 HARs specific for neural tissue. HARs were also enriched for transcription binding sites, 78%, *p* < 0.0001.

Investigators then proceeded to link their data and map 576 HARs to promoter regions in over 700 genes. Of the 132 target genes associated with an OMIM disorder, 28 (*p* = 0.008) included autism spectrum disorder or intellectual disability in man. Not only were HARs interacting with flanking gene promoters, but also the flanking and target genes were found to be particularly sensitive to gene dosage. This suggests that HARs might provide a new cause of dose regulation for highly constrained and haplosensitive genes: the type of genes known to cause autism spectrum disorder, intellectual difficulties, and epileptic encephalopathy. HAR genes were also enriched for neural development, neuronal differentiation, and axonogenesis. More strikingly, they were also enriched for disorders of brain morphology, for autism spectrum disorder, schizophrenia, and, curiously, autonomic function dysfunction.

Analysis of 2100 sibling-matched autism spectrum disorder probands also identified a 6.5-fold increase in de novo copy number variants containing HARs. The authors report that this pattern was only seen in de novo but not inherited copy number variants and estimated that HAR-containing copy number variants may account for up to 1.8% of all autism cases. This multi-method study then built the scientific case for (1) a mutation in HAR 426 affecting the gene *CUX1*; (2) a HAR mutation between *DPYD* and *PTBP2* affecting *PTBP2*; and (3) two separate homozygous HAR mutations near *GPC4*. Finally, by analysing the genomes of consanguineous individuals with autism and looking for damaging mutations inherited from both parents but affecting the same locus (biallelic), they estimated that this may be the causative mechanism in 5% with a similar familial structure. Furthermore, with so many mutations interacting with *SOX2*, they also make a compelling argument for the role of HARs in neurogenesis.

*Doan RN. et al. (2016) Cell 167(2):341*–*354.*

